# Radiolabeled Chalcone Derivatives as Potential Radiotracers for β-Amyloid Plaques Imaging

**DOI:** 10.3390/molecules28073233

**Published:** 2023-04-04

**Authors:** Pier Cesare Capponi, Matteo Mari, Erika Ferrari, Mattia Asti

**Affiliations:** 1 Radiopharmaceutical Chemistry Section, Nuclear Medicine Unit, AUSL-IRCCS di Reggio Emilia, Viale Risorgimento 80, 42122 Reggio Emilia, Italy; 2 Department of Chemical and Geological Sciences, University of Modena and Reggio Emilia, Via G. Campi 103, 41125 Modena, Italy

**Keywords:** α,β-unsaturated carbonyl compounds, Alzheimer’s disease, chalcones, radionuclide, labeling

## Abstract

Natural products often provide a pool of pharmacologically relevant precursors for the development of various drug-related molecules. In this review, the research performed on some radiolabeled chalcone derivatives characterized by the presence of the α-β unsaturated carbonyl functional group as potential radiotracers for the imaging of β-amyloids plaques will be summarized. Chalcones’ structural modifications and chemical approaches which allow their radiolabeling with the most common SPECT (Single Photon Emission Computed Tomography) and PET (Positron Emission Tomography) radionuclides will be described, as well as the state of the art regarding their in vitro binding affinity and in vivo biodistribution and pharmacokinetics in preclinical studies. Moreover, an explanation of the rationale behind their potential utilization as probes for Alzheimer’s disease in nuclear medicine applications will be provided.

## 1. Introduction

**Figure 1:**
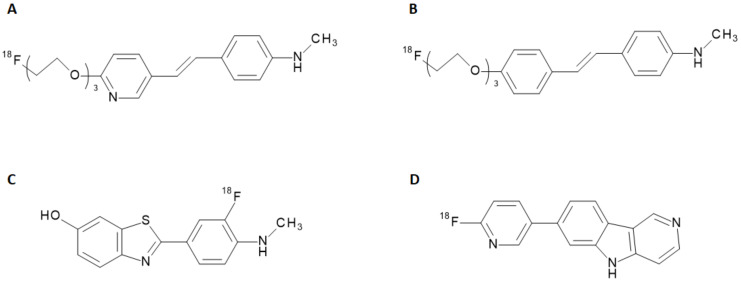
Chemical structure of [^18^F]-florbetapir (Amyvid) (**A**), [^18^F]-florbetaben (Neuraceq) (**B**), [^18^F]-flutemetamol (Vizamyl) (**C**), and [^18^F]-flortaucipir (Tauvid) (**D**).

The potential of new radiopharmaceuticals, along with the technical advancements achieved in nuclear medicine, raised new awareness of the biochemical mechanisms underlying several diseases. Recent progress in the development of radiotracers for Single Photon Emission Computed Tomography (SPECT) and Positron Emission Tomography (PET) have allowed the visualization of metabolic processes unpredictable with other imaging techniques so far. In the neuroimaging field, the advantage offered by the nuclear medicine techniques to non-invasively explore brain disorders makes SPECT and PET diagnostic tools full of promises for early detection of neurodegenerative diseases. During the last decade, the clinical and social need for an accurate diagnosis and monitoring of Alzheimer’s Disease (AD) combined to set up a feasible therapeutic and care approach, prompted the development of new SPECT and PET radiotracers tailored to this purpose. AD is a neurodegenerative disease with insidious onset and progressive impairment of behavioral and cognitive functions, including memory, comprehension, language, attention, reasoning, and judgment. This disease has a high social and economic impact, and there are no definitive treatments to date. Although the accumulation of Aβ-plaques and tau protein fibrils in the brain is acknowledged as being the primary pathological condition of AD [1], the relationship to clinical manifestations as well as the underlying causes of its pathophysiological mechanism have not been elucidated so far [2]. In the last years, many radiolabeled ligands have shown a tropism for Aβ-aggregates and tau proteins and have been employed for preclinical studies or clinical trials [3]. Some of them, such as the stilbene derivatives [^18^F]-florbetapir and [^18^F]-florbetaben (namely, Amyvid™ and Neuraceq™) and the benzothiazole derivative [^18^F]-flutemetamol (namely, Vizamyl™) have finally assumed a clinical relevance, being endowed with a marketing authorization. These radiotracers attest to the abnormal accumulation of Aβ-amyloid plaques in the brain and are thus employed to give early insight into potential AD development [4]. Other molecules, including quinolones, benzimidazoles, and benzimidazole-pyrimidine derivatives, have been investigated for the detection of tau proteins expression [5]. Recently, a radiolabeled benzimidazole-pyrimidine derivativ (namely, [^18^F]-flortaucipir, Tauvid™) has been introduced in the US market as a radiotracer for the detection of tau protein misfolding. The chemical structures of radiotracers used in clinical applications for AD are reported in Figure 1.

**Figure 2:**
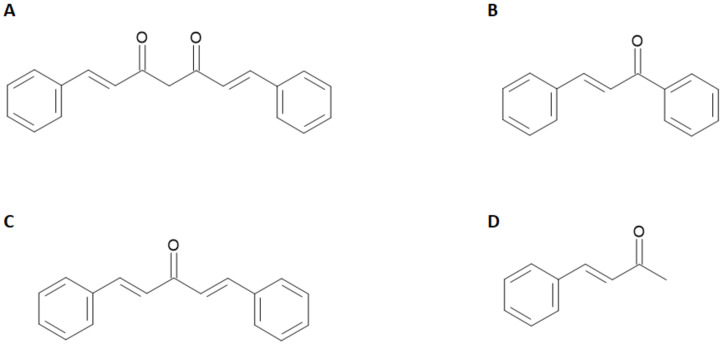
Curcuminod (**A**), chalcone (**B**), dibenzylideneacetone (**C**), and dehydrozingerone (**D**) general structure. Substituents on the aromatic rings provide the specific derivative.

Although the radiopharmaceuticals used hitherto for the detection of pathological pathways associated with AD can provide valuable information to physicians, they are not exempt from some limitations mainly related to their target binding properties. Furthermore, the radiotracers currently in use can detect abnormal Aβ-amyloid deposits only around 5 years before the clinical expression of the disease, showing high affinity for the insoluble Aβ-plaques, but far less affinity for the more toxic soluble ones [6]. It has been suggested that a successful radiopharmaceutical for AD should exhibit features including high blood–brain barrier (BBB) permeation, minimal off-target binding, rapid absorption, and sudden washout from brain tissue as well as low toxicity [4]. All these characteristics are still far from being optimized on the currently used molecules. For these reasons, a parallel branch of research explored the radiolabeling of a heterogeneous family of molecules of natural origin sharing an α,β-unsaturated carbonyl functional group linked to aryl moieties such as curcuminoid, chalcone, dibenzylideneacetone, and dehydrozingerone derivatives. The general chemical structures of these compounds are reported in Figure 2.

For instance, it has been shown that curcumin inhibits the formation and promotes the disaggregation of Aβ-amyloid plaques. Moreover, it attenuates the hyper-phosphorylation of tau protein and enhances its clearance. The applications of radiolabeled curcumin and curcuminoids in medicinal chemistry has been recently reviewed [7,8] but, despite a manifested affinity for amyloid plaques in vitro, none of the radiolabeled probes proved to be a suitable tool for the imaging of AD in vivo. The main reason for this failure is likely due to either the little stability of the curcumin backbone in physiological media and to the inability of the radiolabeled probes to cross the BBB. With the present review, we aim to summarize the studies performed on radiolabeled chalcone derivatives so far attempted. These molecules exhibit a curcumin-like structure but the modifications on the keto-enol group elicit a potential improvement in the stability and hence a potentially better behavior as radiotracers for the detection of Aβ-amyloid plaques.

## 2. Chalcones

The chalcone base structure, namely, [(E)-1,3-diphenyl-2-propene-1-one], is a molecule with two aryl rings separated by α, β unsaturated carbonyl group (Figure 2). Chalcones are generally synthesized in basic conditions (commonly in presence of Na/KOH in EtOH/MeOH solution) by reacting an aromatic methyl ketone with a specific substituted benzaldehyde, as extensively reviewed in [9,10]. This conjugated structure is characterized by a high delocalization of the electrons and a low redox potential that provide remarkable chances of undergoing to electron transfer reactions [11]. Moreover, the unsaturated α-β carbonyl group included between the two aryls moieties is potentially able to react with biological molecules as a Michael acceptor [11]. Chalcones derivatives are present as precursors or components of many natural compounds and have been studied for several medicinal applications such as anti-tumor, anti-ulcer, anti-tuberculosis, anti-inflammatory, and antibacterial. This scaffold is also the constitutive structure of approved drugs; for instance, a synthetic derivative named sofalcone is used as oral gastrointestinal medication [12]. The structure–activity relationship in sofalcone is an example of how the unsaturated α-β carbonyl group can react with nucleophilic groups contained in biological molecules such as thiols. However, despite the structure of these compounds being considered for various biological properties, only very few probes based on the chalcone pharmacophore have been explored as potential radiotracers so far. In the context of AD, chalcones and their derivatives have shown pharmacological potentials against multiple targets by inhibiting both Aβ-fibrils aggregation and the activity of enzymatic systems such as acetylcholinesterase (AChE), butyrylcholinesterase (BuChE), and pseudocholinesterase as well. These enzymes are potentially implicated in the genesis and progression of AD according to the cholinergic hypothesis reported by Pritan et al. [13]. Although the peculiar ability to inhibit these enzymes has been mainly described, it is the chalcones’ additional activity to interact with Aβ-plaques that prompted investigation into their possible application as radiolabeled probes for AD imaging. Despite this, it is known that compounds containing aromatic structures can prevent the assembly of fibrils by disrupting the π-stacking of their hydrophobic *core* [14], however, knowledge of the role played by the functional groups on the chalcone aryl moieties to modulate the binding affinity for Aβ-plaques still remains rather limited and it is the object of the current review.

## 3. Iodine-125, Fluorine-18 and Carbon-11 Labeled Chalcone Derivatives

**Figure 3:**
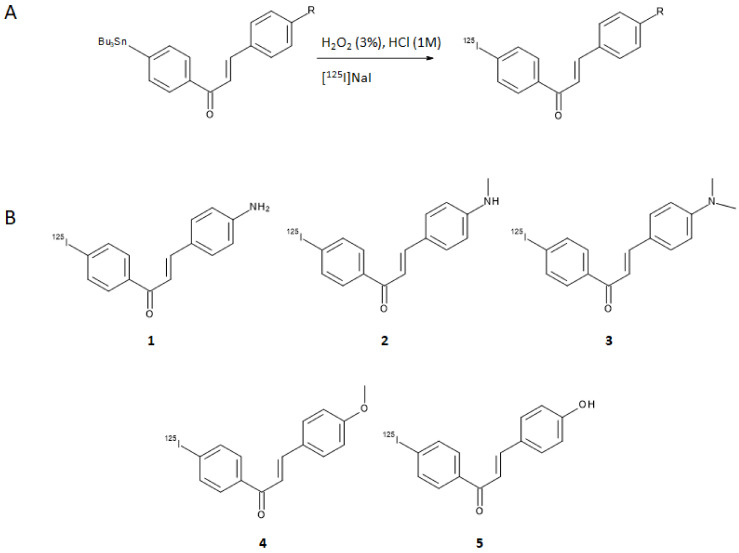
Synthetic pathway for the preparation of general [^125^I]-labeled chalcone derivatives (**A**) and structures of all the derivatives explored in [19] (**B**).

Based on the premises that electron-donating groups such as amino, methylamino, dimethylamino, methoxy, or hydroxy groups play a critical role in the binding affinity to Aβ-aggregates [15,16,17,18], Ono et al. designed a series of five chalcones derivatives containing an electron-donating group in position 4 of the first aromatic ring and an iodine-125 atom in position 4′ of the second aromatic ring [19]. Iodine-125 (E_γ,max_ = 35 keV, t_1/2_ = 59.4 days) is a very suitable radioisotope for pre-clinical research and drugs development purposes, although its emission cannot be harnessed to obtain clear SPECT images. The radioiodinated chalcones were synthesized by iodo-destannylation reaction of the correspondent precursor using hydrogen peroxide as oxidant. Products were obtained in 25–85% radiochemical yields (RCY) with radiochemical purities (RCP) of >95% after HPLC purification and with a theoretical molar activity of 81.4 GBq/μmol. The synthetic pathway and final structures are summarized in Figure 3A,B.

The [^125^I]-chalcone derivatives were tested for their binding affinity to synthetic Aβ(1–42) aggregates and the one containing a dimethylamino moiety ([^125^I]**3**) showed the highest specificity (K_d_ = 4.2 ± 1.1 nm). Additionally, ex vivo assays on brain sections of double transgenic mice used as a model of murine AD pointed out the ability of compound **3** to stain Aβ-amyloid plaques. Binding affinities of all the iodinated chalcones were also evaluated with competitive studies against [^125^I]**3**, obtaining the values reported in Table 1.

**Figure 4:**
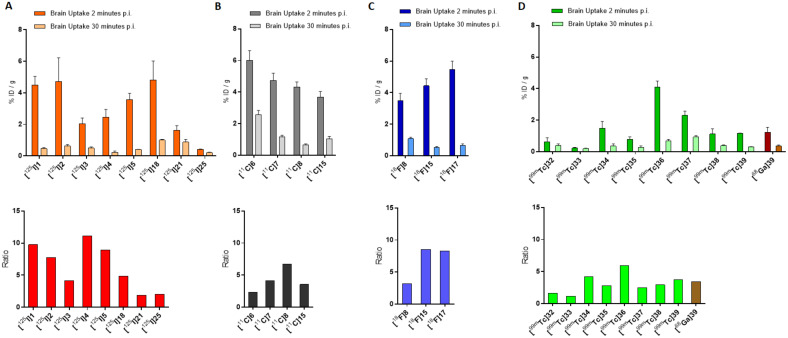
Brain uptakes at 2 and 30 min post injection of [^125^I]-labeled (**A**), [^11^C]-labeled (**B**), [^18^F]-labeled (**C**), [^99m^Tc]- and [^68^Ga]-labeled (**D**) chalcone derivatives in normal mice and the relative ratios.

Finally, biodistribution was evaluated in normal mice for all the correspondent radio-iodinated derivatives. The main prerequisite for an ideal AD radiotracer is a high BBB permeability so as to deliver the necessary activity to the brain and a fast wash-out from the off-target regions to achieve a high signal-to-noise ratio, allowing a good imaging process. This principle will be applied to assess the performance of all the radiolabeled compounds rehearsed in this review. Compounds [^125^I]**1**, [^125^I]**2**, [^125^I]**3**, [^125^I]**4**, and [^125^I]**5** displayed high brain uptakes ranging from 2.0 to 4.7%ID/g at 2 min post-injection (p.i.) and good clearance with residual activity of 0.46, 0.61, 0.49, 0.22, and 0.4% of injected dose per gram of tissue (ID/g) at 30 min p.i., respectively. A comparison of the in vivo behavior of these compounds is summarized in Figure 4A, where the brain uptakes at 2 and 30 min p.i. and the relative ratios are depicted.

Prompted by the results obtained theretofore, the same researchers developed and explored a new series of 12 derivatives where the iodine atom in the position 4′ was replaced with either a fluorine atom or fluoro-pegylated (FPEG) chains of different length (*n* = 1–3) while the amino-, methylamino- or dimethylamino group in position 4 was preserved [20]. The approach was endorsed by the fact that biomolecules contained fluorinated or FPEG groups were effective for some core structures of Aβ-imaging probes [21]. Experiments to determine the in vitro affinity of these chalcone derivatives for Aβ-aggregates were performed with [^125^I] 4-dimethylamino-4′-iodo-chalcone ([^125^I]**3**) as the competing agent, and the results are collected in Table 2.

**Figure 5:**
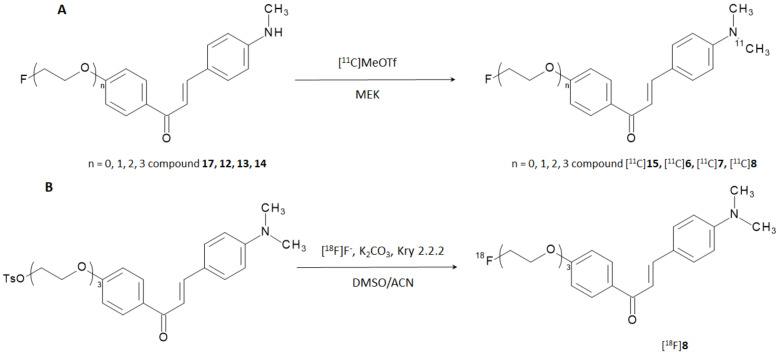
[^11^C]-labeling (**A**) and [^18^F]-labeling (**B**) reaction pathways for the preparation of radiotracers based on F- and FPEG-chalcones structure.

Constant of inhibition (K_i_) values suggested that the affinities for Aβ-aggregates of the compounds were strongly influenced by the substitution at the amino group in position 4 (-NH_2_- < -NHCH_3_ < -N(CH_3_)_2_), while the length of the PEGylated chain in position 4′ influenced the binding only to a minor extent. The results were consistent with those reported in the previous study [19] and the affinity of the most promising candidate (i.e., derivative **7**) is higher than [^125^I]6-iodo-2-(4-dimethylamino)-phenyl-imidazo(1,2-a)pyridine (IMPY),a commonly used competitor for inhibition assays [22,23,24,25]. The study was designed with the additional purpose of obtaining [^18^F]-labeled radiotracers able to provide PET-based imaging of AD but the most promising derivatives (i.e., derivatives **6**, **7**, **8**, and **15**) were first labeled with carbon-11. Fluorine-18 (E_β,max_ = 634 keV, t_1/2_ = 109.8 min) is nowadays the most used positron emitting radionuclide for nuclear medicine examinations, whilst carbon-11 (E_β,max_ = 960 keV, t_1/2_ = 20.3 min) can be introduced in many organic compounds without altering their base structure. Labeling with carbon-11 was carried out by reacting the correspondent N-normethyl precursors **12**, **13**, **14**, and **17** with [^11^C]methyl triflate to give [^11^C]**6**, [^11^C]**7**, [^11^C]**8**, and [^11^C]**15** in a decay corrected yield of 28–35% (Figure 5A). Radiochemical purity (RCP) was >99% with a molar activity of 22–28 GBq/μmol. To evaluate the biodistribution in vivo, the [^11^C]-labeled derivatives were injected in normal ddY mice. In absence of Aβ-amyloids plaques, the experiment was performed to highlight the BBB permeability, brain uptake, and wash-out of the radiotracers. All the derivatives showed an excellent brain uptake already after 2 min p.i. with a brain/blood ratio of 1.64, 1.36, 1.76, and 1.40 for [^11^C]**6**, [^11^C]**7**, [^11^C]**8**, and [^11^C]**15**, respectively. In addition, they displayed a good clearance from the normal brain accounting for 37.6, 21.1, 8.1, and 28.3% of the initial activity persisting in the brain after 60 min from the injection, respectively. The performances of these compounds can be compared in Figure 4B.

Derivative **8** exhibited the highest brain uptake and most rapid wash-out when labeled with carbon-11; hence, it was selected for subsequent labeling with fluorine-18. The reaction was performed through a nucleophilic displacing reaction with [^18^F]F- on a tosylated precursor (Figure 5B). The radiotracer was obtained with a radiochemical yield (RCY) of 45%, RCP > 99%, and molar activity estimated around 35 GBq/μmol.

[^18^F]**8** displayed high uptake in normal mice brain (3.85% ID/g) at 2 min p.i. and was cleared rapidly in the following 60 min showing a final residual activity of 1.07% ID/g. Unfortunately, measurable uptake in bones (3.58% ID/g) was also detected at 60 min p.i. attesting in vivo defluorination. Finally, the capability of staining amyloid plaques in vitro using brain tissue section from Tg2576 transgenic mice and AD patients was carried out by fluorescence and autoradiography. In both cases, compound **8** was able to intensely and specifically stain the expressed Aβ-plaques.

**Figure 6:**
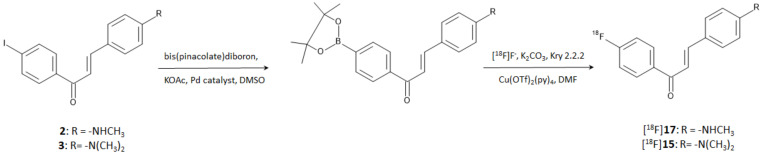
Reaction steps for the direct [^18^F]-fluorination of a chalcone aromatic ring starting from the iodinated precursor.

In a more recent study, Kaide et al. [26] managed to obtain compounds [^18^F]**15** and [^18^F]**17** by direct incorporation of fluorine-18 on the aromatic ring. The reaction was accomplished by harnessing the reactivity of a diboronate compound obtained by the reaction of bis(pinacolate)diboron on the correspondent iodinated precursors. Briefly, the boronate derivatives were used as reagents of a copper-mediated nucleophilic radiofluorination according to the steps shown in Figure 6. Authors proved that iodinated compounds **2** and **3** can be used as starting materials for the direct [^18^F]-labeling of an aromatic ring. The method afforded [^18^F]**15** and [^18^F]**17** with an RCY of 37.0 and 45.0%, respectively, and RCP > 95% after purification by HPLC. The molar activities were 1256 and 466 GBq/μmol, respectively.

The possibility to use compounds [^18^F]**15** and [^18^F]**17** as Aβ imaging probes was evaluated by in vivo pharmacokinetics on normal ddY mice. Moreover, the in vitro binding affinity for Aβ-aggregates and for human brain tissues with AD pathology was tested as well. Both the derivatives attested a higher brain uptake (4.43 and 5.47% ID/g, respectively) at 2 min p.i. than the corresponding radioiodinated chalcones (namely [^125^I]**2** and [^125^I]**3** with an uptake of 2.43 and 3.52% ID/g, respectively) [19] and the dimethylamino-[^18^F]FPEG-chalcone [^18^F]**8** (3.48% ID/g) [20]. Moreover, [^18^F]**15** and [^18^F]**17** displayed higher ratio of radioactivity accumulated at 2 and 30 min p.i. than all the other compared compounds, suggesting a lower non-specific binding to white matter. The same trend was attested when [^18^F]**15** and [^18^F]**17** were compared to the FDA-approved Aβ-imaging agent [^18^F]Florbetapir [27], indicating that these chalcone derivatives are superior to the radiotracer upon in vivo brain accumulation and wash-out. The in vivo behavior of all the cited compounds can be better appreciated and compared in Figure 4A,C where the brain uptakes at 2 and 30 min p.i. and the relative ratios are resumed. Finally, no marked accumulation in the bone was observed at 60 min p.i. (1.65 and 1.86% ID/g, respectively) suggesting that [^18^F]**15** and [^18^F]**17** are more stable toward defluorination in vivo than [^18^F]**8**. In the binding studies using Aβ-aggregates, [^18^F]**15** and [^18^F]**17** exhibited very low Kd values (K_d_ = 4.47 nmol and 6.50 nmol, respectively) indicating high affinity to the target. In the in vitro autoradiography experiment using human brain sections with AD pathology, they intensely and specifically accumulate in regions with Aβ-plaques.

**Figure 7:**
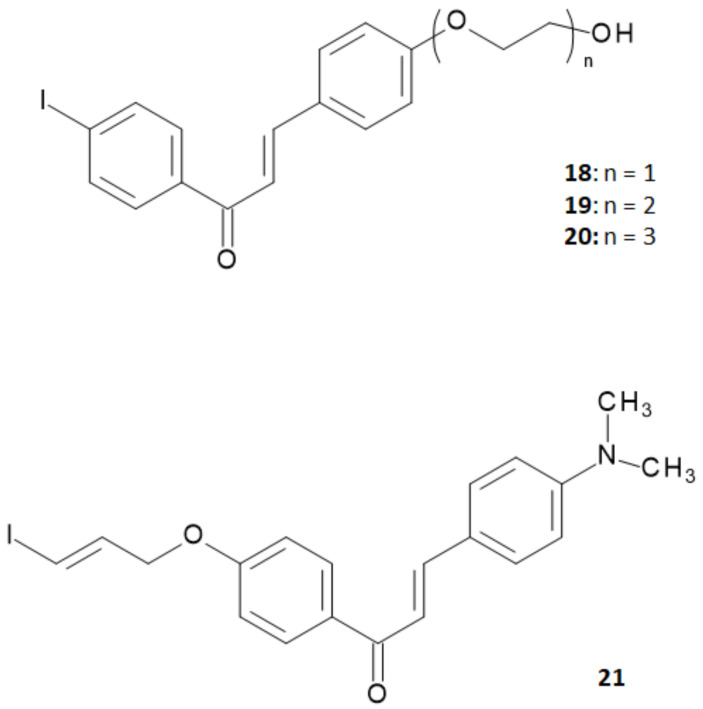
Structure of chalcone derivatives containing ethyleneoxy chains in *para*-position of 3-phenyl ring or an iodoallyloxy group in *para*-position of 1-phenyl ring.

Summarizing the studies hitherto performed, researchers concluded that chalcone derivatives containing a fluoro-pegylated moiety in *para*-position of the 1-phenyl ring showed a sufficiently good in vivo brain uptake and a high in vitro binding affinity to Aβ plaques [20]. On the other hand, it was reported that iodinated flavonoids with a methoxy or oligoethyleneoxy group in *para*-position of the 3-phenyl ring showed high in vivo brain uptake and rapid clearance without significant decrease in the in vitro binding affinity [28,29]. On these bases, Fuchigami and coworkers explored a new series of chalcone derivatives where an iodoallyloxy group in *para*-position of the 1-phenyl ring or ethyleneoxy groups in *para*-position of the 3-phenyl ring were introduced in order to match both of the demands provided by the precedent studies [30]. The structures of this series of derivatives are reported in Figure 7.

To assess the performance of these derivatives as probes able to bind Aβ-amyloid plaques, in vitro binding experiments were performed in suspensions of Aβ (1–42) aggregates using 4-[^125^I]iodo-4′-dimethyamino-calchone ([^125^I]**3**) as the competing agent. Results obtained are summarized in Table 3.

**Figure 8:**
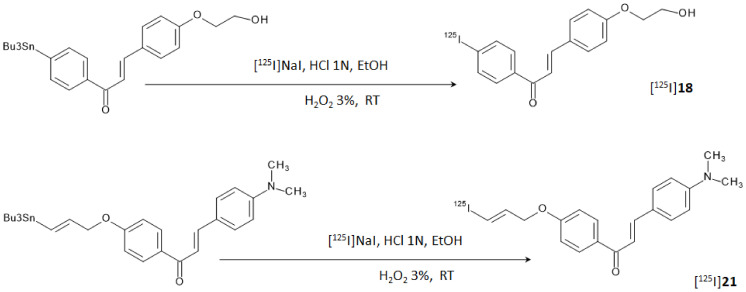
Reaction steps for the labeling of [^125^I]**18** and [^125^I]**21**.

The K_i_ values of the ethyleneoxy-iodochalcone derivatives suggested that the binding affinity of the compound was affected in a non-linear way by the length of the ethyleneoxy group (monoethyleneoxylated derivative **18** > triethyleneoxylated derivative **20** > diethyleneoxylated derivative **19**). On the other hand, the iodoallyloxy-chalcone (compound **21**) showed a binding affinity comparable to [^125^I]**3** that was the most effective derivative until then. Thus, compounds **18** and **21** were selected for further studies and labeled with iodine-125 through an iododestannylation reaction using hydrogen peroxide as oxidant, as reported in Figure 8. [^125^I]**18** and [^125^I]**21** were obtained in radiochemical yields of 66–75% with radiochemical purities of >95% after HPLC purification.

In vitro autoradiography tests using brain sections of Tg2576 mice and the hippocampal section of the human brain attested that only [^125^I]**21** was able to label amyloid plaques as it showed a pattern which effectively matched with the one obtained with thioflavin-S in adjacent brain sections. Conversely, [^125^I]**18** displayed no clear accumulation in the amyloid deposits. This last result was unexpected since the K_i_ obtained for [^125^I]**18** was retained sufficiently low to successfully stain Aβ-amyloid plaques, and it was attributed to the low concentration of radiotracer used for the experiment. On the other hand, biodistribution studies in normal mice showed a high initial uptake (4.82% ID/g at 2 min p.i.) of [^125^I]**18** and a rapid clearance from the brain (1.00% ID/g at 30 min p.i.) whilst [^125^I]**21** showed only a modest uptake (1.62% ID/g at 2 min p.i.) and a relatively slow clearance (0.87% ID/g at 30 min p.i.). These finding suggested the lower stability in vivo of the iodoalliloxy group that rapidly generates hydrophilic metabolites which are unable to cross the BBB. Brain accumulation and clearance of [^125^I]**18** and [^125^I]**21** can be evaluated and compared, along with the other radiotracers herein discussed, in Figure 4. Overall, it can be concluded that the study did not provide significant advancements in the research.

**Figure 9:**
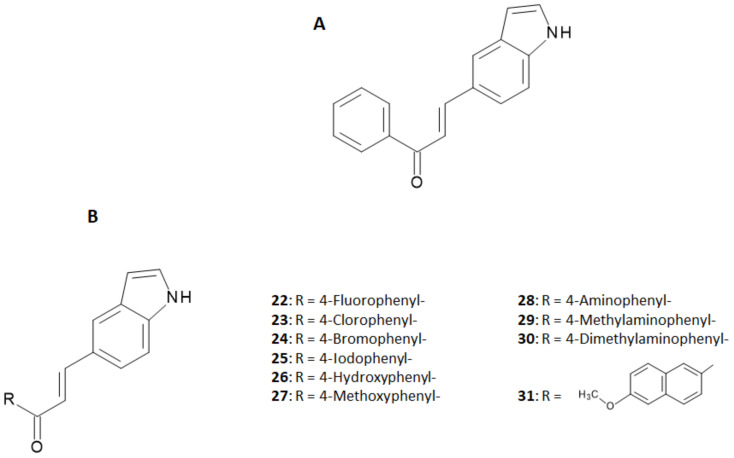
General structure of an indole-chalcone (**A**). Indole-Chalcone derivatives studied by Cui et al. [31] (**B**).

In the development of more useful probes based on the chalcone structure, the research was further extended to chalcone derivatives endowed with an indole ring [31]. The indole structure is often considered as a fused ring of N-methyl- or N,N-dimethylamino groups and the derivatives obtained by this replacement were expected to maintain an affinity for Aβ aggregates as high as the N-alkyl-derivatives studied theretofore [19,20]. In that study, the authors synthesized 10 new indole-chalcone derivatives and evaluated their binding affinity to Aβ_1–42_ aggregates in competition with [^125^I] IMPY [31]. Backbone structures of indole-chalcone and the investigated derivatives are shown in Figure 9, while the binding affinities of the single indole-chalcone derivatives are summarized in Table 4.

As reported in Table 4, the binding affinity result depended on the substituents of the phenyl ring. Halogenation of the para-position enhanced the binding affinity following the trend (F- < Cl- ≈ Br- ≈ I-). Replacement of the halogen with a hydroxyl group dramatically decreased the affinity, while its substitution with a methoxyl group restored the affinity to the level of the halogens. On the other hand, affinity was completely lost when an amino group was placed at the *para*-position, but it increased again with its methylation (-NH_2_- < -NHCH_3_ < -N(CH_3_)_2_), as already reported in precedent studies [19,20]. Enlargement of the aromatic conjugation system improved the affinity as well.

**Figure 10:**
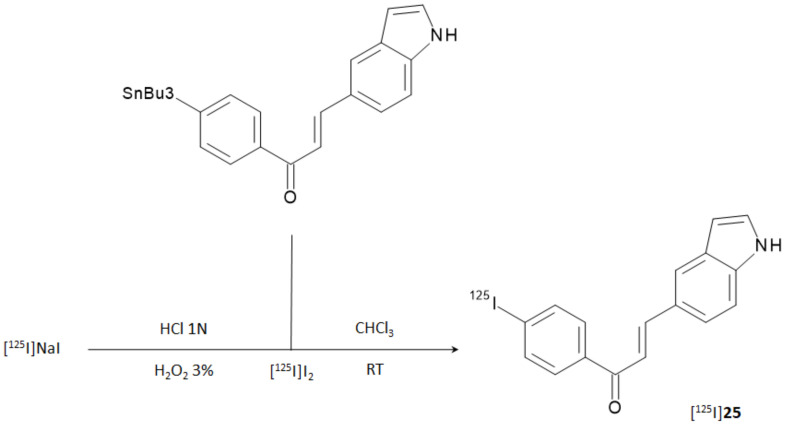
Reaction steps for the labeling of [^125^I]iodophenyl-indole-chalcone ([^125^I]**25**).

Due to the remarkable performance obtained by the indole-chalcone derivative containing the iodophenyl moiety (compound **25**), the researchers carried out further biological evaluations by replacing natural iodine with iodine-125. [^125^I]**25** was prepared from the corresponding tributyltin precursor through a [^125^I]iododestannylation reaction according to Figure 10. HPLC-purified derivative was obtained in 21.1% RCY and with an RCP > 95%. The molar activity was 81.4 GBq/μmol.

In fluorescent staining and autoradiography assays with sections of brain tissue from transgenic mice (C57, APP/PS1, 12 months), [^125^I]**25** was able to stain Aβ-plaques with an effective target to background ratio. The texture of the staining pattern was consistent with that obtained with thioflavin-S in adjacent brain sections. Unfortunately, biodistribution experiments with ddY normal mice showed low brain uptake (0.41% ID/g at 2 min p.i.) and sluggish wash-out (0.20% ID/g at 30 min p.i.), suggesting that further chemical modifications may be necessary for providing a suitable β-amyloid imaging agent based on the indole-chalcone structure. Comparison with the other radiotracers herein discussed can be directly appraised in Figure 4.

## 4. Technetium-99m and Gallium-68 Labeled Chalcone Derivatives

**Figure 11:**
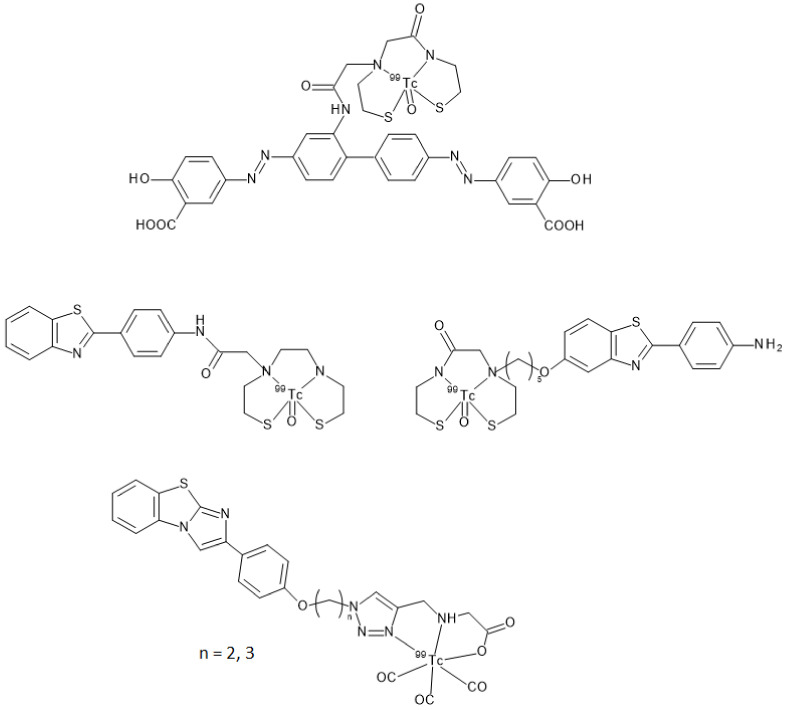
Structures of some [^99m^Tc]-labeled radiotracers able to target β-amyloid plaques obtained by a bifunctional approach as reported in [23,24,25,36].

A different approach was needed for developing and synthesizing innovative chalcone derivatives able to target Aβ-amyloid plaques labeled with technetium-99m. Technetium-99m is a γ-emitting radioisotopes (E_γ_ = 140 keV, 89%) with a moderate half-life of 6 h. The emission is well suited to be detected by medical equipment such as SPECT cameras. It has become the most used radionuclide in diagnostic nuclear medicine mainly because it can be readily produced by commercial ^99^Mo/^99m^Tc generators [32]. Being a metal, technetium-99m can only be inserted in a biological molecule through a complexation reaction with proper chelators covalently linked to a molecular vector [33,34]. Chelators may impart specific features to the structure by enhancing molecular weight and influencing overall charge and lipophilicity. All these characteristics are of paramount importance when crossing the BBB is needed to reach the target. Indeed, Kung et al. showed that a [^99m^Tc]-complex can achieve this aim by reporting that the dopamine transporter imaging agent, [^99m^Tc]TRODAT-1, was useful to detect the loss of dopamine neurons in the basal ganglia associated with Parkinson’s disease [35]. [^99m^Tc]TRODAT-1 was the first example of a [^99m^Tc]-labeled imaging agent able to pass the BBB in which technectium-99m was encapsulated in a bis-amino-bis-thiol chelator (BAT). Prompted by this paradigm, several [^99m^Tc]BAT- or monoamine-monoamide-dithiol (MAMA) radiotracers able to target β-amyloid plaques in the brain of AD patients were proposed (Figure 11) but, unfortunately, no clinical studies followed the pre-clinical development [23,24,25]. Another possible approach entailed the synthesis of a tridentate ligand obtained from a copper catalyzed click reaction on an azide containing benzothiazole. The obtained 2-arylimidazo [2,1-b]benzothiazole ligand was then reacted with with *fac*-[^99m^Tc(CO)_3_(H_2_O)_3_]^+^ to provide the final radiotracer (Figure 11). This derivative proved to be able to cross the BBB with discrete brain accumulation at 2 min p.i. and rapid wash-out [36].

**Figure 12:**
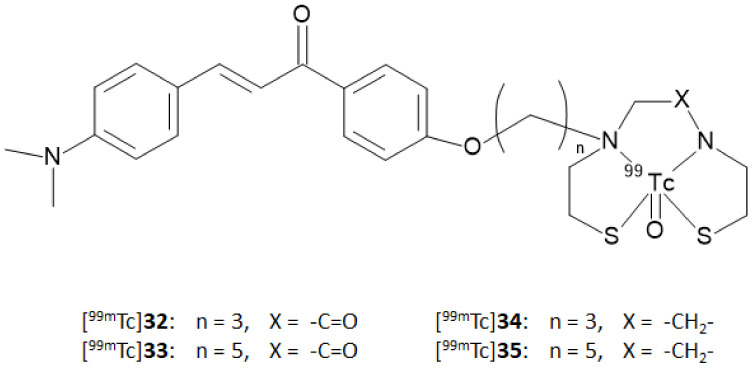
Structures of [^99m^Tc]-labeled BAT and MAMA-chalcone derivatives (compounds ([^99m^Tc]**32**–**35**).

In this context, Ono and coworkers [37] synthesized four chalcone derivatives containing MAMA or BAT chelators linked to the backbones by a three- or five-atom-length alkyl linker (compound **32**, **33**, **34**, and **35**). The ligands were labeled with technetium-99m by a ligand exchange reaction starting from a [^99m^Tc]-glucoheptonate precursor affording the [^99m^Tc]-complexes reported in Figure 12 ([^99m^Tc]**32**–**35**) with an RCP > 95% after HPLC purification.

Binding assays were performed using different concentrations of Aβ(1–42) aggregates. The test was persecuted by measuring the percentage of radioactivity bound to the synthetic Aβ aggregates as a function of Aβ-aggregates concentration. The derivatives were ranked according to this order: [^99m^Tc]**35** > [^99m^Tc]**33** >> [^99m^Tc]**32** ≥ [^99m^Tc]**34**. These findings suggested that the chelator (BAT or MAMA) has little influence on the affinity for Aβ-fibrils, but longer chain derivatives (*n* = 5) have higher affinity than shorter ones (*n* = 3). Although the test was useful to compare the derivatives reported in this study, no competition assays were carried out to calculate the binding affinity constants so no comparison with the previously described [^125^I]-, [^18^F]-, or [^11^C]-chalcone derivatives can be derived. Staining of β-amyloid plaques was also carried out on Tg2576 and wild type mice brain sections using the corresponding rhenium complexes. Many plaques were undoubtedly stained in the Alzheimer’s model mice by all the compounds while only minimum labeling was observed in the wild-type ones. The staining pattern was consistent with that observed with thioflavin-S, proving the specificity of the binding. Despite the promising results obtained by all the four [^99m^Tc]-labeled chalcones in vitro, biodistribution in normal mice attested that only [^99m^Tc]**34** exhibited high brain uptake (1.48% ID/g at 2 min p.i.) and rapid elimination (0.17% ID/g at 60 min p.i.). The authors remarked that the uptake and pharmacokinetics of [^99m^Tc]**34** are superior to any other [^99m^Tc]-labeled probe previously reported in the literature, indicating that this chalcone should be further investigated as a potential radiotracer for β-amyloid imaging. Conversely, [^99m^Tc]**35**, [^99m^Tc]**33**, and [^99m^Tc]**32** showed poor initial uptake (from 0.32 to 0.78% ID/g at 2 min p.i.) and were cleared from the mice brain relatively slowly (0.11–0.16% ID/g at 60 min p.i.). On the whole, the in vitro and in vivo results are quite controversial; however, to date, there are no further published data helpful in solving this conundrum.

**Figure 13:**
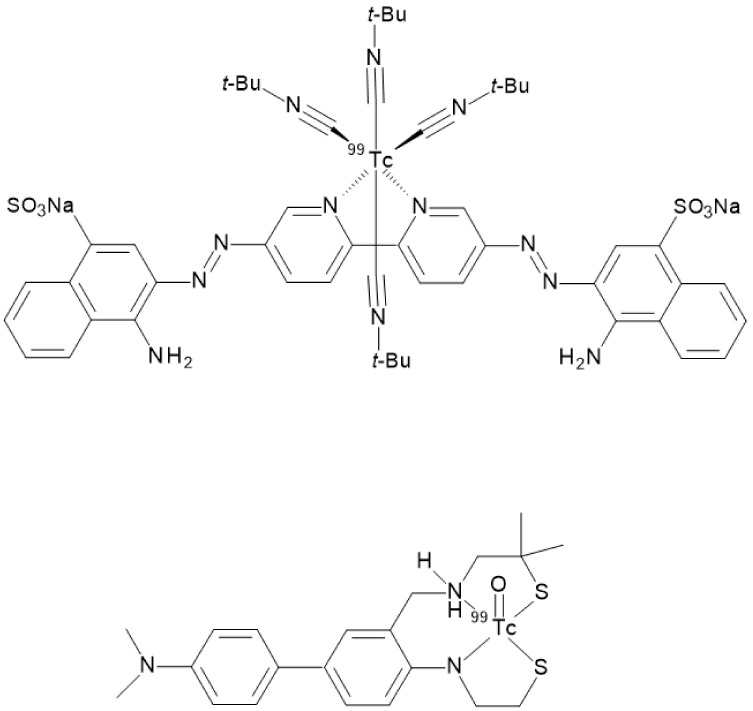
Structures of some [^99m^Tc]-labeled radiotracers able to target β-amyloid plaques obtained by an integrated approach as reported in [22,39].

As already discussed, the passage through the BBB can be influenced by many parameters. Additionally, even small structural changes can have repercussions on the pharmacokinetic and affinity parameters in vivo. The bifunctional approach mentioned so far (i.e., the addition of a proper chelator linked to the targeting vector through a covalent linker) generally leads to an increase in the molecular volume and molecular weight. Given the promising characteristics of the [^99m^Tc]-labeled scaffolds studied theretofore, research groups considered the use of an innovative approach for the functionalization, trying to reduce the influence of the complexation moiety. The so-called integrated approach entails the replacement of parts of the targeting vector with the [^99m^Tc]-chelation moiety in such a way that the latter mimics a part of the base structure, thus triggering minimal changes in size, conformation, and affinity. In the case of radiotracers aiming to target β-amyloids deposits, the steric hindrance of the chelator appended through a bifunctional approach twists the planar shape of the binding agent. The planarity of the vector is a very important feature to fit the planar gap among the Aβ-plaques [3,38] thus, the model of the integrated approach was applied for synthesizing several radiotracers maintaining high affinity to β-amyloid plaques such as the Congo Red and Biphenyl technetium-99m derivatives reported in Figure 13 [22,39].

**Figure 14:**
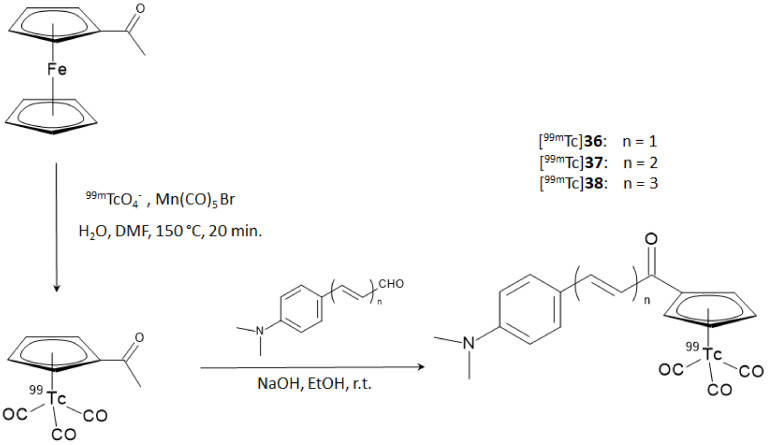
Synthetic steps for the preparation of [Cp^99m^Tc(CO)_3_]-chalcone-mimic derivatives (compounds ([^99m^Tc]**36**–**38**).

On these premises, Li and coworker designed and synthesized a series of chalcone-mimic complexes by replacing one benzene ring with a [^99m^Tc]-tricarbonyl-cyclopentadienyl *core* ([Cp^99m^Tc(CO)_3_]). This “piano stool” organometallic moiety is a neutral 18 electron species that can be coupled to biomolecules with classical organometallic methods without severely affecting their bioactivity [40]. Furthermore, the small size and lipophilicity of the [Cp^99m^Tc(CO)_3_] fragment appeared particularly promising for the labeling of a potential BBB-crossing molecule. In turn, [Cp^99m^Tc(CO)_3_] can be obtained directly from the ^99^Mo/^99m^Tc generator eluate containing [^99m^Tc]TcO_4_^−^ by reaction with sensitive organometallic reagents [40]. The final [^99m^Tc]-labeled chalcone-mimic derivatives and the correspondent rhenium complexes used as reference standards were synthesized with a two steps sequential reaction. In the first step, the [CH_3_COCp^99m^Tc(CO)_3_] or [CH_3_COCpRe(CO)_3_] complexes were obtained by a double ligand transfer reaction, starting from a ferrocene precursor, then the complexes underwent a base-catalyzed Claisen condensation with three aldehydes differing for the length of their π-conjugated system. The overall RCY was 25%, with an RCP > 95% after HPLC purification. The synthetic steps are shown in Figure 14.

The capability of these complexes with respect to fluorescent stains Aβ-plaques was assessed in sections of brain tissue from AD patients and Tg model mice in comparison with normal adults and normal mice brain sections. Specific staining of Aβ-plaques (confirmed by the results obtained with thioflavin-S on adjacent sections) was obtained in Tg mice and AD patients’ brain sections for complexes **37** and **38** with rhenium, whilst no apparent labeling was observed in normal models. Conversely, compound 36 showed feeble signals in both cases, likely suggesting a low affinity. Quantitative binding affinity to Aβ_1–42_ aggregates was hence appraised by an in vitro inhibition assay with [^125^I]IMPY as a competing agent and the obtained Ki are reported in Table 5.

As evident from Table 5, all the rhenium complexes showed a moderate binding affinity for the aggregates but this is still in the range determined for other iodine-25 or fluorine-18 radiolabeled chalcone derivatives. Intriguingly, the Ki value is as high as the length of the π-conjugation (in the order: rhenium complex **38** > **37** > **36**). This trend was also confirmed by experiments computing the bound aggregate activity (%) using the [^99m^Tc]-labeled chalcone-mimic derivatives. The authors inferred that the extension of π-conjugation weakens the distortion of the whole ligand planar configuration, thus enhancing the ligand affinity for the β-amyloid plaques [38]. This consideration was also supported by the comparison of the crystal structures in which the distortion of the dihedral angle between the benzene and cyclopentadienyl planes was more pronounced for complex **36** than complex **37**. Finally, biodistribution experiments were carried out in normal male ICR mice for all the [^99m^Tc]-labeled chalcone-mimic derivatives. Complex [^99m^Tc]**36**, owning the shortest π-conjugation (*n* = 1), exhibited the highest initial brain uptake (4.10% ID/g at 2 min p.i.), followed by [^99m^Tc]**37** (*n* = 2, 2.30% ID/g) and [^99m^Tc]**38** (*n* = 3, 1.1% ID/g). Brain_2min_/brain_60min_ uptake ratios followed the same trend, attesting [^99m^Tc]**36** as the best performing among the three radiotracers. Brain accumulation and clearance of all the [^99m^Tc]-labeled chalcone derivatives discussed so far can be compared in Figure 4D. In general, the early uptake achieved by the [Cp^99m^Tc(CO)_3_] chalcone-mimic derivatives was remarkably higher than the uptake obtained previously by the derivatives resulting from the bifunctional approach. The authors concluded that the integrated approach which used a [Cp^99m^Tc(CO)_3_] *core* as a building block provided encouraging insights for designing radiotracers that mimic the chalcone structure, and so, this path may be pursued for developing useful [^99m^Tc]-labeled radiopharmaceuticals for AD imaging. The efforts to develop [^99m^Tc]-based amyloid plaques targeting radiotracers over the years were also recently reviewed by Takalloobanafshi and coworkers [41].

**Figure 15:**
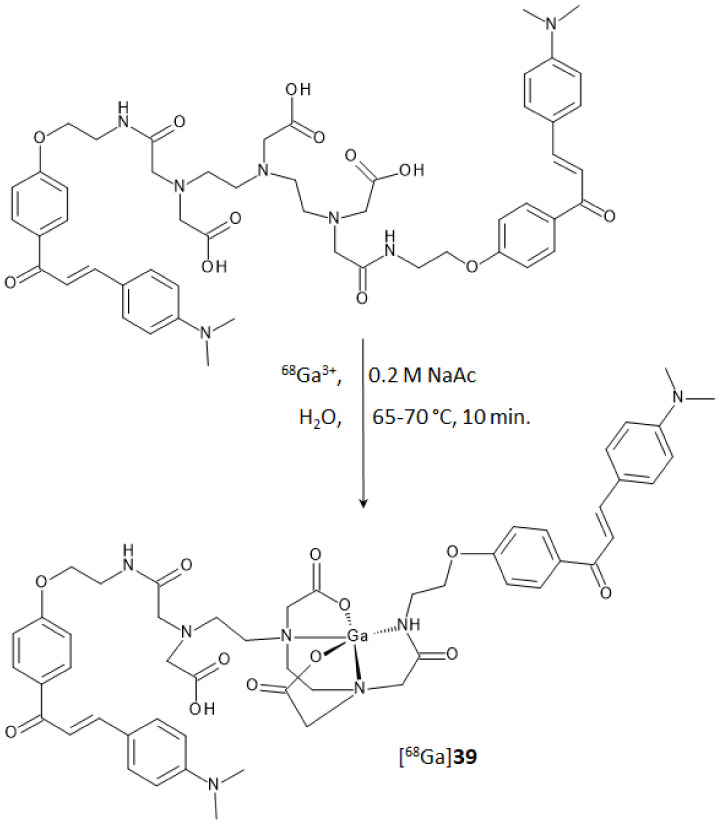
Synthesis of homodimer [^68^Ga]DTPA-chalcone derivative (compound [^68^Ga]**39**).

In the study regarding chalcones, the use of an acyclic chelator such as diethylenetriaminepentaacetic acid (DTPA) was preferred to the more commonly used macrocyclic analogues DOTA or NOTA. In comparison to cyclic chelators, the acyclic ones allow higher complexation rates and flexibility in the metal coordination sphere when mild labeling conditions are used [42]. DTPA is a potential penta-anionic octadentate ligand that was functionalized to achieve a derivative with β-amyloid plaques affinity. Two of the five carboxyl-arms were reacted with a 3-*para*-dimethyamino-chalcone moiety, yielding the homodimer ligand **39** reported in Figure 15. Docking studies on the potential binding sites of the DTPA conjugated chalcone (compound **39**) toward the amyloid protein were recently carried out by Mann et al. [43]. This study revealed docking score values for **39** even higher than some of the currently FDA-approved tracers (i.e., florbetaben, florbetapir, and flutemetamol). In the same paper, **39** was used to complex technetium-99 with a facile single-step synthetic strategy that allowed to isolate, after a solid phase extraction, a potential amyloid-targeted Aβ imaging agent with high yield and purity ([^99m^Tc]**39**). The complex displayed high-Aβ aggregates binding affinity during in vitro evaluation and, notably, brain uptake in normal mice at 2 min p.i. (1.16% ID/g) followed by rapid washout at 30 min p.i. (0.31% ID/g). Brain accumulation and clearance of this derivative were compared with those of the other [^99m^Tc]-labeled chalcones in Figure 4.

A further step in the development of radiotracers derived from chalcones containing a radioactive metal *core* was pursued by obtaining a complex of ligand **39** with gallium-68 [44]. Gallium-68 (E_β,max_ = 1899 keV, 89%, 67.7 min) is a valuable positron emitter that achieved a steady utilization in clinical practice and research in the last years [45,46]. Optimal labeling was achieved in a pH range from 3.5 to 4.5 at a temperature of 65–70 °C. The RCY was around 85% with RCP of 99% and 10 MBq/nmol molar activity. Synthesis of the corresponding natural gallium(III) complex was also performed as the reference standard for the characterization and affinity studies. DTPA coordination sphere and consequent structure of complex [^nat/68^Ga]-**39**, reported in Figure 15, were postulated from a recent review reporting the ORTEP representation of several gallium complexes [47].

Binding studies of [^68^Ga]-**39** were performed on artificially aggregated Aβ_42_-peptides and transformation of the saturation binding to a Scatchard plot resulted in a one-site binding with a K_d_ of 3.46 nm. In the inhibition assay performed with [^68^Ga]-**39** as the competing agent, Ga-**39** showed an inhibition constant comparable to that of compound **3** (K_i_ = 4.18 and 3.64 nmol, respectively). The radioactive fraction bound to Aβ-aggregates was around 10.5%, while blocking studies performed with a 100-fold excess of cold complex or compound **3** displayed an uptake reduction of 80–85%. Finally, to understand the pharmacokinetics of [^68^Ga]-**39** and its applicability as a radiotracer in vivo, biodistribution studies in normal adult male BALB/c mice were performed. The complex showed a good brain uptake of 1.24% ID/g at 2 min p.i. and a rapid wash-out with residual activity of 0.36% ID/g at 30 min p.i. These performances are comparable to those exhibited by the [^99m^Tc]-labeled chalcone derivatives described before (Figure 4D), and to those of [^11^C]-PIB, an established radiotracer for AD imaging [48]. Notably, [^68^Ga]-**39** showed no significant uptake in other non-target organs such as the lungs, spleen, and heart. The authors concluded that despite violating some empirical rules of BBB permeability (for instance, molecular weight generally > 1000/mol), this complex and other chalcone derivatives complexes of radiometals, such as the technectium-99m and gallium-67/68, might afford promising candidates for the imaging of AD and open new avenues for radiopharmaceuticals supply.

## 5. Final Remarks and Future Perspectives

Over the years, a long series of chalcones derivatives was labeled with several radionuclides, and the relationship between structure modifications and functional enhancement was deeply investigated with the purpose of providing useful radiotracers for the early detection of AD. From the radiochemical point of view, sundry strategies have been explored for improving radiolabeling performance, mainly leveraging the cutting-edge paths followed for the most common radionuclides used in nuclear medicine applications. Iodine-125 was introduced directly on activated aryls, following common approaches such as iododestannylation reaction, while fluorine-18 needed tosylated precursors or more recently emerged pathways such as the use of aryl boronic precursors. Labeling with carbon-11 was commonly carried out by reacting the N-normethyl precursors with [^11^C]methyl triflate. Technetium-99m was introduced in a *plethora* of chalcone derivatives, using the bifunctional chelator approach with MAMA, BAT, and DTPA moieties or by an integrated approach, replacing part of the chalcone structure with a cyclopentadienyl group. Finally, a [^68^Ga]-labeled homodimer analogues was synthesized by exploiting the acyclic chelator DTPA. The effect of any structural changes and the impact of using different radionuclides on the final affinity of the radiotracer for the β-amyloid plaques were explored with a mixture of techniques. Usually, affinity studies and binding assays were assessed on synthetic aggregates but in vitro staining of fluorescent chalcone derivatives in the brain sections of mice and human expressing plaques were also performed. Finally, in vivo biodistribution studies were commonly carried out on normal mice to appraise brain uptake and wash-out of the radiotracer examined. If the ratios between the brain uptake at 2 min and 30 min of all the radiotracers summarized in this review are compared (Figure 4), it can be inferred that the molecules carrying a metal radionuclide (such as technectium-99 and gallium-68) generally exhibit lower ratios than the radiohalogen containing ones. The lower performances can be ascribed both to the presence of the chelator that introduces major bias in the chalcone structure, lowering the affinity for the β-amyloid plaques, and to the hydrophilic nature of the chelator itself, which may hinder the passage through the BBB. Unfortunately, none of the described radiolabeled chalcone derivatives have been tested in vivo in mice developing β-amyloid plaques; thus, we lack a convincing proof-of-concept that this family of radiotracers can provide positive results as AD seeking molecules in clinical application. Indeed, the capability of staining amyloid deposits in brain sections of AD-expressing mice and patients ex vivo by autoradiography appeared insufficient to guarantee an effective translation in clinical studies. These latter considerations are the main reason for the stagnation of the research concerning the radiotracers based on the chalcone structure, and should be addressed in an effort to relaunch the interest in the topic.

## 6. Conclusions

The results summarized in this review give insight into new applications of natural molecules containing α-β unsaturated carbonyl functional group as potential new radiopharmaceuticals for early diagnosis of AD. Actually, the chalcone moiety has the potential for further fine-tuning aimed towards developing a suitable PET or SPECT amyloid imaging agent. The structure itself inherits further studies in order to improve affinity and the biodistribution pattern. However, research on radiolabeled chalcone compounds still has a bumpy road ahead, in which the first pivotal step should be the in vivo studies on mice developing β-amyloid plaques.

## Figures and Tables

**Table 1:** Inhibition constants of iodinated chalcone derivatives against 4-[125I]iodo-4′-dimethyamino-calchone ([^125^I]**3**) as adapted from [19].

Label	-R	K_i_ (nm)
**1**	-NH_2_	104.7 ± 12
**2**	-NHCH_3_	6.3 ± 1.6
**3**	-N(CH_3_)_2_	2.6 ± 0.3
**4**	-OCH_3_	6.3 ± 1.7
**5**	-OH	21.4 ± 1.4

**Table 2:** Inhibition constants of fluoro- and FPEG-chalcone derivatives against 4-[125I]iodo-4′-dimethyamino-calchone ([^125^I]**3**) as adapted from [20].

Label	-R_1_	-R_2_	K_i_ (nm)
**6**	FCH_2_CH_2_O-	-N(CH_3_)_2_	45.7 ± 7.1
**7**	F(CH_2_CH_2_O)_2_-	-N(CH_3_)_2_	20.0 ± 2.5
**8**	F(CH_2_CH_2_O)_3_-	-N(CH_3_)_2_	38.9 ± 4.2
**9**	FCH_2_CH_2_O-	-NH_2_	678.9 ± 21.7
**10**	F(CH_2_CH_2_O)_2_-	-NH_2_	1048.0 ± 114.3
**11**	F(CH_2_CH_2_O)_3_-	-NH_2_	790.0 ± 132.1
**12**	FCH_2_CH_2_O-	-NHCH_3_	197.1 ± 58.8
**13**	F(CH_2_CH_2_O)_2_-	-NHCH_3_	216.4 ± 13.8
**14**	F(CH_2_CH_2_O)_3_-	-NHCH_3_	470.9 ± 100.4
**15**	F-	-N(CH_3_)_2_	49.8 ± 6.2
**16**	F-	-NH_2_	663.0 ± 88.3
**17**	F-	-NHCH_3_	234.2 ± 44.0

**Table 3:** Inhibition constants of ethyleneoxy-iodochalcone and iodoallyloxy-chalcone derivatives against 4-[125I]iodo-4′-dimethyamino-calchone ([^125^I]**3**) as adapted from [30].

Label	Structure	-R	K_i_ (nm)
**18**	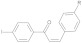	-OCH_2_CH_2_OH	24.0 ± 10.4
**19**	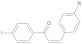	-(OCH_2_CH_2_)_2_OH	127.1 ± 27.3
**20**	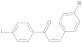	-(OCH_2_CH_2_)_3_OH	87.8 ± 20.3
**21**	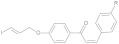	-N(CH_3_)_2_	4.5 ± 1.5

**Table 4:** Inhibition constants of indole-chalcone derivatives against [^125^I] IMPY as adapted from [31].

Label	-R	K_i_ (nm)
**22**	4-Fluorophenyl-	35.06 ± 6.21
**23**	4-Chlorophenyl-	8.43 ± 2.13
**24**	4-Bromophenyl-	8.96 ± 0.92
**25**	4-Iodophenyl-	8.22 ± 1.46
**26**	4-Hydroxyphenyl-	>360
**27**	4-Methoxyphenyl-	8.52 ± 2.15
**28**	4-Aminophenyl-	>1008
**29**	4-Methyaminophenyl-	51.09 ± 0.32
**30**	4-Dimethyaminophenyl	5.17 ± 0.32
**31**	2-methoxynaphthyl-	4.46 ± 0.37

**Table 5:** Inhibition constants of [Cp^99m^Tc(CO)_3_]-chalcone-mimic derivatives against [^125^I] IMPY as adapted from [38].

Label	π-Conjugation	K_i_ (nm)
**36**	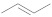	899 ± 78
**37**	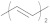	211 ± 19
**38**	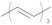	108 ± 16

## Data Availability

Not applicable.
